# Rigid forceps and excimer laser use for complex inferior cava filter retrieval: a preliminary quantitative analysis of available evidence

**DOI:** 10.1186/s42155-022-00311-4

**Published:** 2022-07-08

**Authors:** Qian Yu, Patrick Tran, Deepak Iyer, Subhash Gutti, Davey Li, Xin Li, Rakesh Navuluri, Thong Van Ha, Osman Ahmed

**Affiliations:** 1 Department of Radiology, University of Chicago Medical Center, University of Chicago, Chicago, Illinois 60637 USA; 2 School of Medicine, George Washington University, Washington D.C., 20052 USA; 3 College of Medicine, University of Illinois at Chicago, Chicago, IL 60612 USA; 4 Department of Radiology, University of Pennsylvania, Philadelphia, Pennsylvania 19104 USA

**Keywords:** Filter retrieval, Forceps, Inferior vena cava filter, Laser

## Abstract

**Purpose:**

The present study aims to evaluate the safety and efficacy of advanced inferior vena cava filter (IVCF) retrieval using laser assistance compared with forceps via systematic review and quantitative aggregation of available data.

**Methods:**

Pubmed and Embase were queried from establishment to September 2021. Original studies with a sample size ≥ 5 that reported at least one primary outcome of patients who underwent laser- or forceps-assisted IVCF retrieval were included. Primary outcomes included technical success and complication rates. Baseline characteristics were extracted: age, sex, presence of filter thrombus, strut penetration, previous retrieval attempt, filter dwell time, fluoroscopy time, and filter type. Complications were categorized by type and severity. Categorical data was pooled and evaluated with chi-square or Fisher exact tests.

**Results:**

From the 16 included studies, a total of 673 and 368 patients underwent laser- and forceps-assisted IVCF retrieval, respectively. Successful retrieval was achieved in 98.1 and 93.7% patients from the laser and forceps groups, respectively (*p* < 0.001). Major complication rates (1.6 vs 2.1%, *p* = 0.629) and risk of injury to cava or adjacent organs (1.0 vs 1.4%, *p* = 0.534) were similar between the two groups. A higher proportion of filters from the laser arm were closed-cell design (75.4 vs 68.1%, *p* = 0.020).

**Conclusion:**

Based on limited available evidence, forceps- and laser-assisted complex IVCF retrievals were equally safe. The use of laser sheath is associated with a higher retrieval rate than forceps alone, though the baseline characteristics of two cohorts were not controlled. Future large-scale case-controlled comparative studies with longer clinical follow-up are warranted.

**Supplementary Information:**

The online version contains supplementary material available at 10.1186/s42155-022-00311-4.

## Introduction

The past decade has seen a steady decline in placement with concurrent increase in retrieval of inferior vena cava filters (IVCFs), likely a consequence of heightened medicolegal scrutiny and an increased awareness of complications from prolonged implantation (Ahmed et al. 2017, 2018). While most IVCFs can be successfully retrieved via conventional technique with snare and sheath, filters that are embedded in the caval wall, tilted, and/or of a permanent design are often recalcitrant to standard techniques and may result in retrieval failure. These chronic indwelling filters may also lead to caval thrombosis, perforation, adjacent organ injury, and patient anxiety. Advanced, non-standard approaches to IVCF removal include a variety of techniques, including wire loop methods, balloon dissection, rigid forceps, and/or excimer laser ablation (Merritt et al. 2022). Among these complex IVCF retrieval techniques, rigid forceps and excimer laser provide operators the ability to dissect an endothelialized filter from the caval wall through blunt dissection and thermal ablation, respectively. However, both techniques demand operator proficiency due to risk of damaging the surrounding structures. To date, only a few institutions represented in the published literature are equipped with both and have reported outcomes. Knowledge of IVCF retrieval and complication rates associated with their use is also limited by scarce evidence and potentially subject to experience bias when evaluated on a single institution basis. As a result, analysis of aggregate data from existing knowledge on forceps and laser techniques may shed further light on procedural safety and effectiveness. The purpose of the present study was to therefore characterize the safety and efficacy of these two dissection approaches via systematic review and quantitative analysis.

## Method

Institutional Review Board Approval was not required for this review, as no human or animal subject was involved, and it complies with the Preferred Reporting Items for Systematic Reviews and Meta-analysis Statement (PRISMA) (Moher et al. 2010).

### Literature screening

**Fig. 1:**
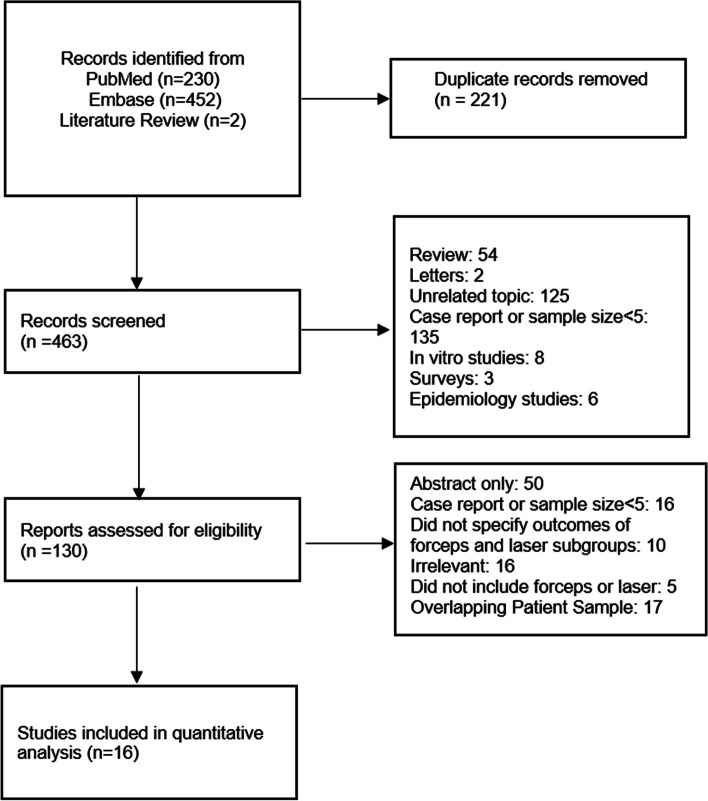
Flow diagram of literature screening

A PICO-based (population/patient, intervention, comparison, outcome) search strategy was conducted using Pubmed and Embase from establishment to September 2021. The following keyword terms were used: (“complex”or“advanced”or“forceps”or“laser”or“excimer”or“endobronchial”) and “inferior vena cava” and “filter”. The following inclusion criteria were adopted: a) studies including patients with IVCF retrieved with excimer laser or endobronchial forceps; b) studies with at least one clinical outcome reported including technical success or complication rates; c) studies with sample sizes of at least 5 patients. A study was excluded if the following criteria were met: a) non-human studies; b) case reports or studies with sample size <5 patients (forceps or laser subgroup); c) absence of original data (letter, editorial, commentary, and review); d) studies that did not specify the outcomes of the forceps- or laser-assisted subgroup separately from the rest; e) epidemiological studies; f) preclinical studies. Endnote 20 (Clarivate Analytics, Philadelphia, Pennsylvania) was used to identify and remove duplicates. Articles were initially screened based on titles, abstracts, and keywords, followed by a comprehensive review of full text of the remaining studies. A detailed screening process is depicted in Fig. 1.

### Data acquisition and statistical analysis

The primary outcomes were technical success and complication rates, which were pooled. Reasons for failure were reviewed. Complication rates were categorized based on type, and severity was divided as major and minor based on Society of Interventional Radiology Guidelines (Sacks 2003). Major complications are defined as complications 1) requiring therapy or minor hospitalization, 2) requiring major therapy, unplanned increase in level of care, or prolonged hospitalization, 3) permanent adverse sequelae, 4) and death. Minor complications are defined as complications 1) requiring no therapy or resulting in no consequence and 2) requiring nominal therapy but without consequence or overnight observation only. Baseline characteristics were retrieved, including author, publication year, region, sample size, filter type, filter dwelling type, procedure information (fluoroscopy time and radiation exposure), and follow-up length. Data was retrieved by three authors (QY, DI, and SG); any discordance was resolved by discussion. All quantitative analyses were performed with Stata 15.1 (STATA Corp., College Station, TX, USA). The pooled results of the forceps and laser subgroups were compared with chi-square or Fisher exact tests for categorical variables.

## Results

### Baseline characteristics

Among the initial 463 search results, a total of 16 studies met criteria for the present analysis (Fig. 1), comprising of 673 and 368 patients who underwent laser- and forceps-assisted IVCF retrieval, respectively (Ahmed et al. 2020; Al-Hakim et al. 2014; Anzai et al. 2021; Avery et al. 2015; Bundy et al. 2021; Chen et al. 2019; Desai et al. 2020; Dowell et al. 2016; Joe et al. 2019; Kuo et al. 2020; Stavropoulos et al. 2015; Tavri et al. 2019; von Stempel et al. 2019; Lian et al. 2019; Scher et al. 2015; Posham et al. 2017) (Table 1). Outcomes of laser-assisted retrieval were reported in three studies; forceps-assisted retrieval were reported in twelve studies; only one study reported outcomes of both modalities (Ahmed et al. 2020). Based on available data for the laser and forceps groups, the average age was 49 and 46 years with a female proportion of 55.0 and 55.2%, respectively. A higher proportion of IVCF retrieved with laser-assistance presented with a thrombosed filter prior to retrieval (11.8 vs 4.0%). The percentage of IVCFs with caval penetration into adjacent organs was 36.8% in the forceps arm. By contrast, only one study from laser-assisted group elaborated on the proportion of IVCFs with cava wall penetration, reporting that 16.2% patients presented with pain from struts damaging adjacent organ; among those patients from the laser cohort with reported data, there were penetrations involving small bowel (*n* = 21), pancreas (*n* = 3), liver (*n* = 1), psoas muscle (*n* = 6), vertebral body (*n* = 10), and aorta (*n* = 8) (Kuo et al. 2020). The proportion of caval penetration in the forceps cohort with pain was not reported. The proportion of patients in who failed previous attempts at IVCF retrieval was 85.7% in the forceps cohort and 88.9% in the laser cohort. Patients who underwent laser-assisted retrieval had a longer average IVCF dwelling time (52.3 vs 18.5 month) and a higher proportion of closed-cell design (75.4 vs 68.1%). The average intraoperative fluoroscopy time was similar between two groups (23.3 vs 28.2 min).

**Table 1:** Pooled baseline characteristics and clinical outcomes of included study

	**Laser**	**Forceps**	***P*-value**
**Sample Size**	673	368	NA
**Age**	49 (500)	47.3 (250)	NA
**%Female**	55.0% (275/500)	55.2% (138/250)	0.9586
**Filter Thrombus**	11.8% (59/500)	4.0% (6/150)	0.001
**Strut Penetration into Adjacent Organ**	16.2% (81/500)^a^	36.8% (68/185)	<0.001
**Previous Retrieval Attempt**	88.9% (594/668)	85.7% (240/280)	0.166
**Dwell Time (month)**	52.3 (143)	18.5 (225)	NA
**Fluoroscopy (min)**	23.3 (143)	28.2 (201)	NA
**% Closed-cell Design**	75.4% (456/605)	68.1% (175/257)	0.020
**Successful Retrieval**	98.1% (636/648)	93.7% (342/365)	0.002
**Major Iatrogenic Injury to Cava or Adjacent Organ**	1.0% (7/673)	1.4% (5/318)	0.534
**Major Complication**	1.6% (11/673)	2.1% (7/338)	0.629

Parentheses indicate included patient sample size of selected parameter

*NA* Not applicable

^a^Symptomatic cases from strut penetration into adjacent organs only

### Technical success

The pooled success rate for IVCF retrieval was 98.1% among patients who underwent laser-assisted retrieval, which was higher than the forceps cohort (93.7%, *p* < 0.001, Table 1). According to Kuo et al., three patients with 2 Optease (Cordis Endovascular, NJ, USA) and 1 Trapease (Cordis Endovascular, CA, USA) filters, respectively, presented with bulky calcified thrombi within the filter that could not be safely removed using thrombectomy and were subsequently too large for the laser sheath to engage. In the forceps group, Avery et al. reported two cases in which IVCFs were not able to be retrieved due to caval wall penetration and attachment to adjacent organs; in four cases, initial retrieval was unsuccessful and an additional attempt was carried out on a separate day (S.Table 1).

### Complication

The pooled major complication was low per category (S.Table 2). No procedure related mortality was reported. There were four cases of caval injury during laser-sheath IVCF retrieval, requiring balloon tamponade and/or stent-graft placement, while such complications occurred in five patients who underwent forceps-assisted retrieval. Adjacent organ injury occurred in three cases of the laser group due to the removal of struts penetrating into adjacent arteries: One patient developed renal infarction after removing a Celect filter (Cook Medical, Bloomington, IN)with its tip penetrating the renal artery; one case of arterial bleeding occurred after removing a Greenfield filter (Boston Scientific, Watertown, MA) with leg adjacent to a branch of the sacral artery; another case of IVC hemorrhage and gastroduodenal artery (GDA) bleeding involving a penetrating, tilted, and fractured Option filter (Argon Medical, Plano, TX) adjacent to the GDA occurred as a sequela of asymmetric advancement of the laser sheath in an attempt to free the filter, but resulted in excessive damage to the cava wall. Other major complications include one case of iatrogenic strut embolization into the atrium (forceps), iatrogenic thromboembolism due to thrombus detaching from the filter (2 lasers, 1 forceps), deep vein thrombosis in lower extremities (forceps), sepsis from removing a filter with strut penetrating into the bowel (laser), major access hematoma requiring overnight observation (laser), and a filter that was stuck at the access site requiring surgical retrieval (forceps). No statistically significant difference was seen between laser and forceps in terms of the rate of major injury to the vena cava or adjacent organs (1.0 vs 1.4%, *p* = 0.534, Table 1). The pooled major complication rates were also similar (1.6 vs 2.1%, *p* = 0.629). Other complications that were minor or not further elaborated upon by original authors included minor renal vein hematoma, postoperative chest pain, postoperative hypoxia, and minor caval pseudoaneurysm.

## Discussion

According to the present quantitative analysis of published literature, the use of laser and forceps assistance in complex IVCF retrieval was highly successful at rates of 98.1 and 93.7%, respectively. Meanwhile, their pooled major complication rates were less than 2%, highlighting the safety of both maneuvers at centers with expertise. While both are highly effective, the use of these two techniques are contingent on the condition of the indwelling IVCF and its anatomical complexity. For instance, forceps are versatile. They can be useful for separating the apex of filters from the caval wall, capture tilted apices, remove fractured fragments, and release filter parts incorporated into the caval wall. In comparison, lasers can dissect the tissue adhering to the filter struts when the apex can be engaged. In practice, these two devices are often complementary, and the application of one device does not preclude the other. In scenarios in which both techniques may be feasible, operator preference/comfort and availability is a primary determinant for which technique is utilized.

Compared to conventional technique with snare and sheath, forceps offers operators greater versatility in filter manipulation. For filters with tilted apices, forceps can be useful in disengaging the apex of a filter away from the caval wall, allowing direct filter retrieval or creating a window for conventional technique with snare and sheath. In cases where struts are embedded, forceps can provide more traction than traditional snares. For thrombosed and endothelialized filters, forceps allow the operator to dissect adhesions between filter and caval wall. In addition, forceps can be useful for retrieval of fractured or embolized filter fragments (Stavropoulos et al. 2015). From an economical perspective, forceps can be re-used multiple times before physically wearing out. Thus, the higher number of published studies describing forceps-assisted IVCF retrieval can be attributed to its versatility, low-cost, and high availability.

By contrast, in cases when filter struts are embedded but the apex is able to be engaged, laser can reduce the pulling force required to dissect the endothelialized tissue attached to the struts and facilitate filter retrieval. The average filter dwelling time was longer among patients who required laser-assisted retrieval (52.3 vs 18.5 months), which allowed more extensive filter endothelialization to occur and therefore necessitated laser for dissection. Furthermore, a higher percentage of filters retrieved with laser appeared to be of closed-cell design (75.4 vs. 68.1%, *p* = 0.02). The filter designs, such as TrapEase (Cordis Endovascular, CA, USA) and OptEase (Cordis Endovascular, NJ, USA) filters are typically more difficult to extract as they are associated with increased surface area contact with the cava in comparison to their open-cell counterparts, resulting in extensive endothelization and fibrosis of the IVC (Desai et al. 2020; Rimon et al. 2011; Oliva et al. 2005; Chick et al. 2016; Kuo et al. 2012). Excimer laser offers the option of dissecting this fibrotic tissue with high precision. Due to the nature of study designs, included cohort studies on laser-assisted IVCF retrieval included both technically difficult closed-cell IVCFs and straightforward cases where the filter was well-positioned without strut penetration. It is unclear if the heterogenous mixture of “easy” and “difficult” filters create an overall cohort similar to the forceps cohort. Further, an unreported percentage of patients who underwent laser-assisted IVCF retrieval were also managed with forceps, as these two modalities are not mutually exclusive in practice. As such, despite the statistical significance of higher technical success rate of laser-assisted IVCF retrieval (*p* < 0.001), potential variation in patient’s baseline characteristics and selection bias undermine the clinical value of comparative analysis in the present study. Nonetheless, the findings of this study tout the safety and efficacy of both of these techniques when employed in the proper clinical scenario.

While the paucity of data on laser-assisted IVCF retrieval often raises concern regarding its safety profile owing to the use of invasive thermal ablation, the pooled data of 673 patients from the present study suggests that laser sheath did not confer a higher risk to surrounding organs compared to forceps. The pooled rate of major injury to the cava and adjacent organs were 1.0% (7/673) among patients who underwent laser-assisted IVCF retrieval, with similar rates in the forceps-assisted group (1.4%, 5/318). Furthermore, previous literature also suggests that most iatrogenic caval pseudoaneurysms during complex filter retrieval with forceps and laser resolve upon balloon tamponade without the need for covered stent placement, suggesting a low clinical significance (Hadied et al. 2021). In the present study, no difference was observed regarding pooled major complication rates between the two groups (2.1 vs 1.6%, *p* = 0.629). These findings suggest that laser-sheath photoablation can be safely implemented in complex IVCF retrieval.

The first detailed report on forceps-assisted IVCF retrieval in human dates to 2006, when Stavropoulos et al. described its use for a Recovery filter (Bard Peripheral Vascular, Tempe, AZ) with the tip embedded in the IVC (Stavropoulos et al. 2006). By contrast, the debut of laser sheath technique did not occur until later in 2010, when Kuo et al. successfully retrieved a Günther-Tulip (Cook Medical, Bloomington, IN) filter by circumferentially ablating the dense fibrotic tissue between the struts and caval endothelium (Kuo and Cupp 2010). Compared to relatively more available evidence on the use of forceps, the majority of peer-reviewed articles on laser-assisted IVCF retrieval are limited to case reports; only four institutions have published data with a sample size at least five patients (Ahmed et al. 2020; Desai et al. 2020; Kuo et al. 2020; von Stempel et al. 2019). In addition to its relatively late introduction to clinical practice, its higher cost and off-label use status also hindered the laser-sheath technique from gaining wider acceptance. Moreover, laser requires safety training and certification along with a 250-volt physical outlet connection, while forceps are less costly and can be re-sterilized for repeated use. Cost-effective analyses are necessary to evaluate the understand the added cost of laser or forceps for complex filter retrieval. Interestingly, despite the relative abundance of cohort studies on forceps-assisted IVCF retrieval, the pooled sample size of the laser-sheath arm is one-fold higher (673 vs 368). As the laser-based device recently obtained breakthrough designation and received FDA authorization for removal of IVCFs, an increase of evidence from additional institutions can be expected to be published in the near future.

The present study has several limitations. First, there are variations of patient baseline characteristics. Few studies reported the type of filter, positioning of the filter, previous retrieval attempts, or degree of strut penetration into the cava, which determine the choice of laser, forceps, or potentially a combination of both. Similarly, a lack of standardization exists with respect to which technique should be used after standard filter removal failure. Compared to laser, the use of forceps is more versatile, which degrades the value of this study serving as a head-to-head comparison as the clinical problem and applications are different. Second, the number of included studies and the total sample size were limited. Only four studies were available in the laser sheath arm, whereas the total number of patients from the forceps group is small. Due to this heterogeneity, implementing chi-square or Fisher exact tests to compare simple pooled outcomes may not be accurate. Third, most studies are of relatively low quality given their retrospective and single-arm design. Several studies were excluded from the present analysis because outcomes of the laser and forceps subgroups were not specified separately (Kuo et al. 2012; Zhou et al. 2012; Kleedehn et al. 2019). While publishing these separate subgroup outcomes will increase the sample size for meta-analysis, high-quality case-controlled comparative studies are also warranted to evaluate laser- and forceps-assisted complex IVCF retrieval in selected scenarios. Finally, most of the included studies were technically oriented, only reporting the immediate clinical results. These studies rarely investigated if complications occurred, or if symptomatic relief was achieved in the post-operative period. Long-term follow-up data is necessary to shed light on the durability of clinical benefits provided by each advanced IVCF retrieval technique.

## Conclusion

Based on the limited available evidence, both forceps and excimer laser were equally safe and effective strategies for complex IVC filter retrieval. The use of laser sheath is associated with a higher retrieval rate than forceps alone, though the present quantitative analysis is limited by heterogeneity. Future case-controlled, comparative studies with large sample sizes and clinical follow-up are warranted.

## Supplementary Information

**Additional file 1: Supplement Table 1.** Baseline Characteristic and Success Retrieval Rates of Included Studies. **Supplement Table 2.** Complication Rates and Iatrogenic Filter Fracture.

## Data Availability

The datasets generated and/or analyzed during the current study are from published manuscripts.

## Abbreviations

IVCF: Inferior vena cava filter
GDA: Gastroduodenal artery
