# Supplementary material for: Rigid forceps and excimer laser use for complex inferior cava filter retrieval: a preliminary quantitative analysis of available evidence
Source: CVIR Endovasc. 2022 Jul 8;5:33. doi: 10.1186/s42155-022-00311-4 (PMC9270549; doi:10.1186/s42155-022-00311-4)
Supplement: Supplementary file 1 — Additional file 1: Supplement Table 1. Baseline Characteristic and Success Retrieval Rates of Included Studies. Supplement Table 2. Complication Rates and Iatrogenic Filter Fracture. [file 42155_2022_311_MOESM1_ESM.docx]

| **Study/Year** | **Technique** | **Region** | **Sample Size** | **Age (mean)** | **M: F** | **Thrombus** | **Caval**  **Embedment** | **Previous Retrieval Attempt** | **Dwelling Time**  **(month)** | **Operating Parameters （mean）** | **Filter Type** | **Indication of Laser or Forceps Retrieval** | **Combined Technique** | **Success Rate** | **Reason of Failure** | **Follow Up (month)** |
| --- | --- | --- | --- | --- | --- | --- | --- | --- | --- | --- | --- | --- | --- | --- | --- | --- |
| Ahmed 2020 | Laser | USA | 5 | NA | NA | NA | NA | NA | NA | NA | NA | Failure of prior standard retrieval or filter related complications | NA | 5/5 | None | NA |
| Kuo 2020 | Laser | USA | 500 | 49 | 225:275 | 59/500 | 81/500 (pain due to penetration), 49 of which involves adjacent organ injury. | 500/500 | 50.9 | NA | Gunther-Tulip: 202  Option: 40  Celect: 35  Celect Platinum: 7  Denali: 12  G2 Meridian: 5  Optease:65  Trapease: 29  Titanium Greenfield: 25  Stainnless Steel Greenfield: 24  Simon-Nitinol: 8 | Failed standard retrieval and high force | NA | 497/500 | Three patients with bulky calcified thrombus refractory to thrombectomy within cylindrical-shaped filter components, which is too large to be captured by the laser sheath apparatus (Optease x2, Trapease x1). | NA |
| Desai 2020 | Laser | USA | 143 | NA | NA | NA | NA | 69/143 | 57.6 | Fluoroscopy: 23.3min;  Radiation dose: 1239mGy | Gunther Tulip: 67  Option 13  Option Elite: 7  OptEase: 33  TrapEase: 7  Simon-Nitinol: 5  Celect: 12  ALN: 1  G2: 1  Greenfield: 2 | Standard retrievals are attempted first. Loop wire or endoscopic forceps are considered. Laser is used when strut incorporation hinders filter retrieval. | NA | 134/143 | NA | NA |
| Von Stempel | Laser | UK | 25 | NA | NA | NA | 13/25 (uncertain degree) | 25/25 | NA | NA | NA | Standard loop-snare failed due to IVC embedment and/or filter is tilted. | 3 laser + forceps | NA | NA | NA |
| Ahmed 2020 | Forceps | USA | 49 | NA | NA | NA | NA | NA | NA | NA | NA | Failure of prior standard retrieval or filter related complications | NA | 47/49 | Non-specified. But both were successfully retrieved on another day. | NA |
| Al Hakim 2014 | Forceps | USA | 17 | NA | NA | 0/17 | NA | NA | NA | NA | Non-permanent | NA | NA | 13/17 | NA | NA |
| Anzai 2021 | Forceps | Japan | 27 | NA | NA | NA | NA | 27/27 | NA | NA | NA | Failure of prior standard retrieval | 10 forceps + sling technique | 27/27 | None | NA |
| Avery | Forceps | Australia | 13 | 51 | 7: 6 | 1/13 | 13/13 (uncertain degree) | 8/13 | 14.0 | Fluoroscopy: 13.2min | G2X: 13  Celect: 7 | Failure of prior standard retrieval or filter related complications | NA | 11/13 | One G2X filter with struct penetration into abdominal aorta; the filter hook cannot be fully freed from the side wall to be grasped with forceps. One Celect filter with deep penetration and anchored into the psoas muscle. | 3-6 (range) |
| Bundy | Forceps | USA | 5 | 54.2 | 2:3 | NA | NA | NA | 112.1 | Fluoroscopy: 130.6;  Air-Kerma: 5442mGy | Simon-Nitinol: 5 | NA | forceps, laser, balloon, hangman; forceps only; forceps, laser, hangman; forceps, laser, balloon; forceps hangman. | 5/5 | None | NA |
| Chen | Forceps | China | 8 | NA | NA | NA | 8/8 | 8/8 | NA | NA | NA | Filter was densely embedded in the IVC wall and failed standard technique. | NA | 8/8 | None | NA |
| Dowell | Forceps | USA | 6 | NA | NA | NA | NA | 6/6 | NA | NA | NA | Failed standard techniques. | NA. | 6/6 | NA | NA |
| Joe | Forceps | USA | 11 | NA | NA | NA | 11/11 (Bowel Penetration) | NA | NA | NA | NA | NA | Forceps+  loop snare in one case | 11/11 | NA | 1-3 (range) |
| Lian | Forceps | China | 27 | 57.9 | 14:13 | NA | NA | 27/27 | 10.9 | Fluoroscopy: 25.9min | Gunther Tulip:12  Celect: 9  Aegisy: 4  OptEase:2 | Failed standard retrieval. | NA | 27/27 | None | NA |
| Posham | Forceps | USA | 25 | 55.1 | 13:12 | NA | NA | 25/25 | NA | Fluoroscopy time: 10.4min (median);  Dose area product: 79.6 Gy · cm2 (median) | Option: 25 | Previous retrieval failure. | NA | 25/25 | None | NA |
| Scher | Forceps | USA | 6 | 50 | 2:4 | 2/6 | NA | NA | 38.4 | Fluoroscopy: 35.0min | OptEase: 3  Trapease:3 | NA | 5 Forceps and snare | 6/6 | None | NA |
| Stavropoulos | Forceps | USA | 114 | 43 | 37:77 | 3/114 | Grade 0: 12  Grade 1: 14  Grade 2: 42  Grade 3: 44 | 79/114 | 15.5 | fluoroscopy: 24min. | G2: 33  Celect: 31  Gunther Tulip: 13  Eclipse: 11  Recovery: 10  G2X: 8  Option: 6  OptEase: 1  ALN: 1 | NA | NA | 109/114 | Two G2 filter, one Recovery, one Celect, one Eclipse. | At least 1 month in 85 patients |
| Tavri | Forceps | USA | 60 | 49.3 | 37: 23 | NA | Grade 0: 0  Grade 1: 3  Grade 2: 44  Grade 3: 13 | 60/60 | 18.8 | Fluoroscopy: 33.2min. | Option: 33  Celect: 8  Gunther Tulip: 9  G2: 4  Eclipse: 3  OptEase: 2  Simon Nitinol: 1 | Failure of prior retrieval | 5 forceps + snare loop | 58/60 | One Option filter had migration of fractured limb into right atrium requiring a second attempt.  One patient had extreme discomfort requiring general anesthesia on another day. | NA |

**Supplement Table 1**: Baseline Characteristic and Success Retrieval Rates of Included Studies.

| **Study** | **Group** | **Strut Embolization** | **Thromboembolism** | **Caval Injury** | **Adjacent organ Injury** | **Sepsis** | **Access Hemorrhage/**  **Injury** | **Total Major Complications** | **Other Complications** | **Total Reported Complications** |
| --- | --- | --- | --- | --- | --- | --- | --- | --- | --- | --- |
| Ahmed | Laser | 0 | 0 | 0 | 0 | 0 | 0 | 0 | Unknown | 0 |
| Kuo | Laser | 0 | 2 | 4 | 3 | 1 | 0 | 10 | Unknown | 10 |
| Desai | Laser | 0 | 0 | 0 | 0 | 0 | 1 | 1 | Unknown | 1 |
| Von Stempson | Laser | 0 | 0 | 0 | 0 | 0 | 0 | 0 | Unknown | 0 |
| Ahmed | Forceps | 1 | 1 | 0 | 0 | 0 | 0 | 0 | Unknown | 2 |
| Al Hakim | Forceps | 0 | 0 | 2 | 0 | 0 | 0 | 2 |  | 2 |
| Anzai | Forceps | 0 | 0 | 0 | 0 | 0 | 0 | 0 | 1 renal vein hematoma, minor | 2 |
| Avery | Forceps | 0 | 0 | 0 | 0 | 0 | 0 | 0 | Chest pain | 1 |
| Bundy | Forceps | 0 | 0 | 0 | 0 | 0 | 0 | 0 | Caval injury, minor | 1 |
| Chen | Forceps | 0 | 0 | 0 | 0 | 0 | Unknown | Unknown | 3 hematoma, unknown severity | 3 |
| Dowell | Forceps | Unknown | Unknown | Unknown | Unknown | Unknown | Unknown | Unknown | Unknown | Unknown |
| Joe | Forceps | 0 | 0 | 0 | 0 | 0 | 0 | 0 | 1 deoxygenation | 1 |
| Lian | Forceps | 0 | 0 | 0 | 0 | 0 | 0 | 0 | 2 minor cava injury | 2 |
| Posham | Forceps | 0 | 1 | 0 | 0 | 0 | 1 | 2 | Unknown | 2 |
| Scher | Forceps | 0 | 0 | 0 | 0 | 0 | 0 | 0 | Unknown | 0 |
| Stavropoulos | Forceps | 0 | 0 | 1 | 0 | 0 | 0 | 1 | 3 | 4 |
| Tavri | Forceps | 0 | 0 | 2 | 0 | 0 | 0 | 2 | Unknown | 2 |

**Supplement Table 2**: Complication Rates and Iatrogenic Filter Fracture.
